# The availability of novelty sweets within high school localities

**DOI:** 10.1038/sj.bdj.2016.412

**Published:** 2016-06-10

**Authors:** A. Aljawad, M. Z. Morgan, J. S. Rees, R. Fairchild

**Affiliations:** 1 Clinical Research Student, Cardiff Metropolitan University, Cardiff, ,; 2 Senior Lecturer in Dental Public Health, Applied Clinical Research and Public Health, College of Biomedical and Life Sciences, Cardiff University, School of Dentistry, ,; 3 Senior Lecturer in Nutrition, Cardiff Metropolitan University, Cardiff, ,; 4 Professor of Restorative Dentistry, Cardiff University School of Dentistry, Cardiff, ,

**Keywords:** Nutrition and diet in dentistry, Paediatric dentistry

## Abstract

**Background** Reducing sugar consumption is a primary focus of current global public health policy. Achieving 5% of total energy from free sugars will be difficult acknowledging the concentration of free sugars in sugar sweetened beverages, confectionery and as hidden sugars in many savoury items. The expansion of the novelty sweet market in the UK has significant implications for children and young adults as they contribute to dental caries, dental erosion and obesity.

**Objective** To identify the most available types of novelty sweets within the high school fringe in Cardiff, UK and to assess their price range and where and how they were displayed in shops.

**Subjects and methods** Shops within a ten minute walking distance around five purposively selected high schools in the Cardiff aea representing different levels of deprivation were visited. Shops in Cardiff city centre and three supermarkets were also visited to identify the most commonly available novelty sweets.

**Results** The ten most popular novelty sweets identified in these scoping visits were (in descending order): Brain Licker, Push Pop, Juicy Drop, Lickedy Lips, Big Baby Pop, Vimto candy spray, Toxic Waste, Tango candy spray, Brain Blasterz Bitz and Mega Mouth candy spray. Novelty sweets were located on low shelves which were accessible to all age-groups in 73% (14 out of 19) of the shops. Novelty sweets were displayed in the checkout area in 37% (seven out of 19) shops. The price of the top ten novelty sweets ranged from 39p to £1.

**Conclusion** A wide range of acidic and sugary novelty sweets were easily accessible and priced within pocket money range. Those personnel involved in delivering dental and wider health education or health promotion need to be aware of recent developments in children's confectionery. The potential effects of these novelty sweets on both general and dental health require further investigation.

## Key Points

- Highlights the growth in the novelty sweets market.
- These sweets are acidic and sweet and therefore can lead to both dental caries and dental erosion.
- The high sugar content may also contribute to overnutrition and overweight or obesity.
- The design of these sweets, in resealable containers, encourages repeated consumption which is detrimental to oral health.

## Introduction

Dental caries, dental erosion and obesity are non-communicable diseases common in UK children.^1,2,3^ Research has established that diet is one of the major aetiological factors in the development of dental caries, dental erosion^4,5,6,7,8^ and obesity among children.^9,10,11^

There is a particularly strong relationship between eating foods high in 'free' sugars and dental caries^7,12,13^ The term free sugars refers to all mono and disaccharides added to foods by the manufacturer, cook or consumer, plus sugars naturally present in honey, fruit juices and syrups.^14^

Reducing sugar consumption to 5% of total energy from free sugars is a primary focus of current global public health policy.^14,15^ Achieving this goal will be difficult acknowledging the concentration of free sugars in children's diets. It has been estimated that sugars, preserves and confectionery contribute 21% free sugars to the total energy of 11-18-year-olds in the UK, with 5% being attributed specifically to sugar confectionery.^16^

In the UK decennial surveys of children's teeth have been carried out since 1973.^2^ Encouragingly, since 1983 there has been a downward trend in dental caries experience among all age-groups taking part. However, the prevalence remains high for this largely preventable disease. In 2013, nearly a half (46%) of 15-year-olds and a third (34%) of 12-year-olds had 'obvious decay experience' in their permanent teeth. Worryingly, there are wide inequalities in experience, with all age groups eligible for free school meals (that is, lower income families) having greater experience of dental caries.^2^

Epidemiological studies have highlighted that frequent consumption of acidic foods and/or drinks can lead to the development of dental erosion.^8,17^ Many of these acidic products contain high levels of free sugars which also contribute to the development of dental caries.^18^

The development of tooth surface loss (TSL) at an early age in the deciduous and the mixed dentition is becoming an increasing concern for the dental profession with erosion being the primary cause. The most recent National Child Dental Health Survey reported an increase TSL for all age-groups taking part between 2003 and 2013. For example, in 12-year-olds TSL in incisors increased from 12% to 24% and from 30% to 38% in buccal and lingual surfaces respectively; the increase in molar teeth was from 19% to 25%.^2^

Recent systematic reviews have documented that the consumption of foods high in free sugars can lead to an increase in overweight and obesity.^9,19^ According to the 2011 Health Surveys for England, Scotland and Wales, the percentage of children aged 2–15 years who were obese was 5.5% for boys and 7.2% for girls. Furthermore, the percentages for children either obese or overweight were 22% and 28% for boys and girls respectively.^3^

Over the last decade sour and novelty sweets have continued to gain popularity in the UK.^20^ Novelty sweets are characterised by being resealable, both sweet and sour tasting, are usually brightly coloured, resemble or can be used as toys and are sold at pocket money prices.^21^ The marketing of novelty sweets is mainly directed towards children who are the primary consumers of confectionery in the UK.^18^ A focus group study of novelty sweets highlighted that 9-10-year-olds thought novelty sweets were aimed at children older than themselves.^21^ This older age group also have more spending power according to pocket money surveys.^22^ Sour sweets were first introduced in the late 1970s by adding a sour flavoured coating which contained a mixture of simple organic acids such as citric, malic and tartaric, to the surface of the sweet. Sour sweets, incorporating novelty sweets, a more recent development, have grown in market share and social acceptability. For example in the UK, in 2015 Haribo was the leading social brand food company according to their Fast Moving Consumer Goods ranking.^23^

Novelty sweets are of particular concern because they contain both high levels of free sugars and acids. Furthermore, their product design facilitates regular frequency of consumption (many are resealable). Consequently, they have the potential to cause dental caries and dental erosion and for children to consume extra 'empty calories' which could lead to the development of overweight or obesity. It is because of these concerns relating to oral and general health that it is important to address free sugars, including confectionery consumption, as a part of an overall health promotion programme.^1,15^

To date, studies on the health implications of novelty sweets are limited, addressing only the pH, neutralisable acidity and enamel loss associated with their consumption^24,25,26^ and their general availability to children.^22^ The objective of this study was to build on existing research by identifying the most available types of novelty sweets, assess their price range and where and how they were displayed in shops.

## Materials and methods

**Figure 1:**
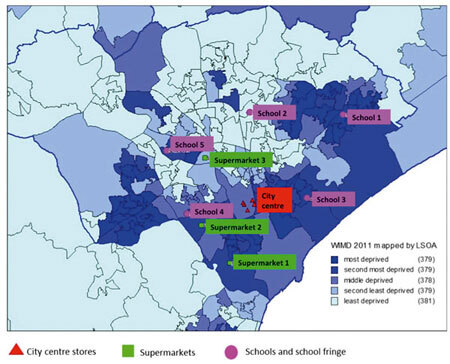
Reference locations (five schools, five city centre stores and three supermarkets) Cardiff Unitary Authority, by quintiles of deprivation, WIMD 2011

Scoping visits were undertaken to determine the varieties of novelty sweets available within the high school fringe^29^ of selected schools in Cardiff, UK. A list of the most available novelty sweets was created by visiting purposively selected city centre stores (non-limited to sweet shops; it is known that some fashion stores stock sweets), shops located near five high schools and three supermarkets from the wider Cardiff conurbation (Fig. 1).

High schools (children aged 11–18 years) were purposively selected to represent a cross-section of the socio-economic characteristics of the city using the Welsh Index of Multiple Deprivation^28^ to inform this process. Five high schools were selected, one in each deprivation quintile.

Shops located within the school fringe were visited, each shop within a radius of ten minutes' walking distance. A stopwatch was used to estimate the walking distance of each shop selling novelty sweets from the chosen schools. The school fringe was determined as the area in close proximity to schools according to the definition provided by Sinclair and Winkler^27^ 'where children can buy items while walking to and from school and during lunch time'.

Shops around the schools were visited just before children left school at the end of the school day (14:00-15:00 hrs); for the other retail outlets visits were conducted during weekdays. At all visits novelty sweets available for sale were noted. In addition, the retail prices and the location of display were recorded with regard to the height of the shelves and the proximity to the check out. Post visit an assessment was made of the difference in availability of novelty sweets in relation to deprivation. Ethical approval for this study was granted by the Dental School Research Ethics Committee (DSREC reference 15/41a).

Data was analysed using SPSS v20 (IBM Corporation, Chicago, USA). Analysis of data included descriptive statistics, incorporating frequency distributions and cross tabulations. MapInfo v10 (Pitney Bowes, New York, USA) was used to represent WIMD, store and school location data.

## Results

A total of 68 stores were visited; 19 of these stores sold at least one novelty sweet type. In total 84 novelty sweets were identified, but this included repeats. However, 38 unique novelty sweet varieties were available in the 19 stores (Table 1). School 3, in the most deprived area, had the largest percentage of shops selling novelty sweets; this was also apparent within the city centre, where at each location 50%, five out of ten shops sold them (Table 1). In addition more varieties of novelty sweets were sold around these two locations, 16 varieties in close proximity to school 3 and 17 varieties within the selected city centre shops. Furthermore school 2 in the least deprived area had no shops around the school fringe selling novelty sweets (out of the 11 visited), as was the case for school 5 which bordered the second least deprived area out of ten shops visited (Table 1). Both schools 1 and 4 which were in the second most and middle deprived areas of Cardiff respectively had 33% (4/12) of shops selling novelty sweets. The shops surrounding school 1 stocked 15 varieties of different novelty sweets, compared with 11 types in shops close to school 4. Only one of the three visited supermarkets sold novelty sweets; however this was the only supermarket situated within a school fringe of school 4, in the middle deprived area of the city, which stocked five types of novelty sweets (Table 1).

**Table 1:** Summary of types and price range of novelty sweets in shops within school fringes

SETTING	WIMD (2011) deprivation quintile for school location	No. of visited shops	No. of shops selling sweets	Types of Novelty sweets	Price range £
School 1	Second most deprived	12	4	15	0.10-1.49
School 2	Least deprived	11	0	None	–
School 3	Most deprived	10	5	16	0.39-1.00
School 4	Middle deprived	12	4	11	0.39-0.99
School 5	On border with second least deprived	10	0	None	–
City centre	–	10	5	17	0.39-0.99
3 supermarkets	–	3	1	5	0.39-2.99

**Figure 2:**
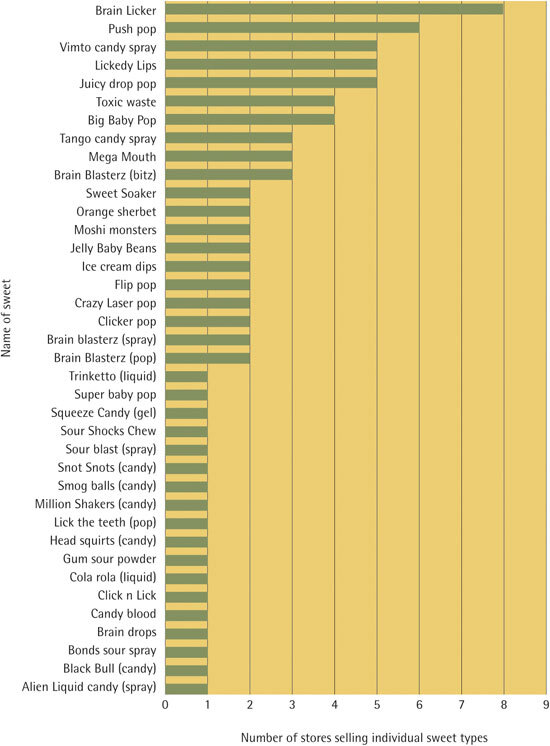
Prevalence in 21 stores, out of the 70 visited, stocking one or more novelty sweets

A frequency distribution of the prevalence of the novelty sweets is presented in Figure 2; the most frequently available sweet variety was Brain Licker, available in eight separate shops. At the other end of the distribution there were 18 unique sweet varieties, including Alien Liquid Candy, Lick the Teeth, Snot Shots and Sour Shocks Chew, which were each available in one shop only.

**Figure 3:**
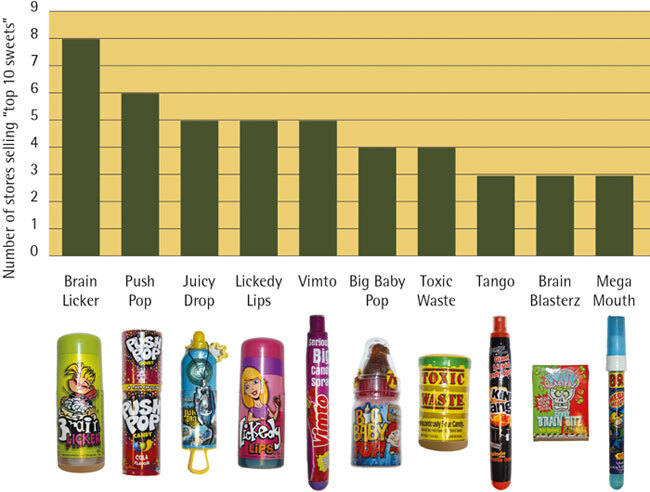
Availability of novelty sweets, for the ten most frequently identified types across 19 Cardiff stores

A visual representation of the ten most available novelty sweets in the Cardiff area is presented in Figure 3 and these were (in descending order):

- Brain Licker
- Push Pop
- Juicy Drop
- Lickedy Lips
- Big Baby Pop
- Vimto candy spray
- Toxic Waste
- Tango candy spray
- Brain Blasterz Bitz
- Mega Mouth candy spray.

**Figure 4:**
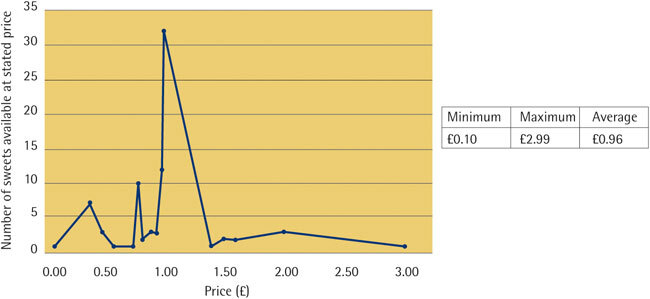
Price distribution of all 84 novelty sweets (including repeats)

The average price of the 38 unique novelty sweet varieties was £0.96 with a range from £0.10 (for Sour Shocks Chew) to £2.99 (for Candy Blood). Thirty-two of the 84 sweet types were priced at £1.00 (Fig. 4). In addition, the novelty sweets were displayed on low shelves (under 136 cm) in 74% (14 out of 19) of the shops, which means that they were accessible to all age groups. Furthermore in 37% (7 of 19) of the shops, novelty sweets were displayed in the checkout area (the remainder were displayed in dedicated confectionery aisles).

The novelty sweets were categorised into seven main types reflecting their textural properties, that is, gels, sprays, liquids and hard candy. The most available product formulations were solid lollipops (for example, Baby Pop), liquid spray (for example, Vimto candy spray), liquids (for example, Brain Licker) and solid candy (for example, Toxic Waste, Table 2).

**Table 2:** Sweets categorised by form

Consistency	Form of sweet	Number (including repeats)
Liquid	Liquid	17
Liquid	Spray	18
Gel	Gel	1
Solid	Candy	17
Solid	Lollipop	22
Solid	Lollipop & powder	6
Solid	Powder	3
**Total**		**84**

## Discussion

From the results of the present study, the most popular novelty sweets identified in the Cardiff area were (in descending order) Brain Licker, Push Pop, Juicy Drop Pop, Lickedy Lips, Big Baby Pop, Vimto candy spray, Toxic Waste, Tango candy spray, Brain Blasterz Bitz and Mega Mouth candy spray. Previous studies in the UK^24,25^ and the Netherlands^26^ investigating novelty sweets also reported on Brain Licker, Juicy Drop Pop, Big Baby Pop and Mega Mouth. The prevalence of novelty sweets as part of the larger sour candy market is also noted by authors in the USA.^29^ The continuing presence of these products indicates good market penetration. The results of the present study showed the availability of a wider range of novelty sweets than previously which may be explained by the recent expansion of this confectionery range in the UK.^20^ For example, Lickedy Lips is a version of Brain Licker which was not included in the previous studies.^24,25,26^ Lickedy Lips appears to be marketed towards girls, in terms of its packaging (a lipstick holder) and colour (pink). The quantification and ranking of the novelty sweets identified in this research could provide a focus for future studies.

Notably, it was found that some stores (n = 6) with multiple outlets sell novelty sweets only in shops in close proximity to schools. These results support the view that targeted marketing of these sweets is being used as a strategy by these stores.^27^

It was also observed in the present study that the availability of novelty sweets was greater in the most deprived areas. The increased availability of sweets in deprived areas may be because they are a palatable low cost source of energy. This result supports previous findings in the USA and the UK.^27,30,31^

The price of the available types of novelty sweets in the visited shops was in the range of £0.10-£2.99, while the price range of the most common novelty sweets was in the range of £0.39-£1.00 with an average price of £0.96. This range of prices is likely to be affordable to children in the UK where the average weekly pocket money for children was reported as £6.20 in 2013.^22^ Children could therefore buy several novelty sweets each week, with potential effects on the general and oral health of children.^7,8,19^ It has been reported that only one in four children in the UK looks for healthy choices in food when shopping.^32^

Novelty sweets were displayed on relatively low shelves and the checkout area, at heights accessible to UK children. This confirms that physical engagement is widely used by retailers in child-targeted marketing.^27^

## Conclusion

A wide range of novelty sweets was available to high school children in the Cardiff area. There appeared to be a relationship between the level of deprivation and the availability of novelty sweets. Furthermore the prices of this type of confectionery were well within the reported pocket money range available to children.

The recent lobby by Public Health England^33^ and others for a sugar tax on sugar sweetened beverages (SSBs) was successful in March 2016.^34^ Limiting the consumption of SSBs should help reduce the overall contribution of free sugars to the national diet but consideration also needs to be awarded to others foods high in sugar, including confectionery, in order to bring about the 5% of total energy goal.^14^

A more holistic approach to change could draw upon the UK national salt reduction campaign. This was coordinated by the UK Food Standards Agency and involved a consumer education programme relating to salt consumption and voluntary salt reduction targets for the food industry. These have achieved significant reductions in salt intakes over the last ten years.^35,36^

The study confirms that novelty sweets are widely available, both in terms of location and cost, to children in Cardiff. It also confirms that this is an expanding market in terms of product diversification and market penetration. Those personnel involved in delivering dental and wider health education or health promotion need to be aware of current trends in children's confectionery. Therefore, the effects of novelty sweets on the general and dental health of children should be further investigated.
